# Gut Microbiota, Leaky Gut, and Autoimmune Diseases

**DOI:** 10.3389/fimmu.2022.946248

**Published:** 2022-06-27

**Authors:** Anna Christovich, Xin M. Luo

**Affiliations:** ^1^ Department of Biological Systems Engineering, Virginia Tech, Blacksburg, VA, United States; ^2^ Department of Biomedical Sciences and Pathobiology, Virginia Tech, Blacksburg, VA, United States

**Keywords:** gut microbiota, leaky gut, systemic lupus erythematosus, type 1 diabetes, multiple sclerosis

## Abstract

With the rising prevalence of autoimmune diseases, the role of the environment, specifically the gut microbiota, in disease development has grown to be a major area of study. Recent advances show a relationship and possible cause and effect between the gut microbiota and the initiation or exacerbation of autoimmune diseases. Furthermore, microbial dysbiosis and leaky gut are frequent phenomena in both human autoimmune diseases and the murine autoimmunity models. This review will focus on literature in recent years concerning the gut microbiota and leaky gut in relation to the autoimmune diseases, including systemic lupus erythematosus, type 1 diabetes, and multiple sclerosis.

## Introduction

While once believed to be driven predominantly by genetics, environmental factors and interactions between the environment and genetics are now considered to be major contributors to autoimmunity. It is imperative to obtain a better understanding of these factors to address the rising incidence of autoimmune diseases, develop appropriate therapies, and possibly suggest lifestyle changes. Previously, we discussed the ability of leaky gut to act as an environmental trigger of autoimmunity when genetic susceptibility exists (1). We also discussed the gut microbiota’s ability to modulate intestinal permeability, leading to perturbation or amelioration of disease depending on the bacteria’s pathogenicity. Hence, the microbiota was suggested as a controllable factor to alter the disease course. Here, we expand on these ideas by specifically focusing on recent advances in our understanding of the gut microbiota and leaky gut in three autoimmune diseases.

## Systemic Lupus Erythematosus

### Background

Systemic lupus erythematosus (SLE) is an autoimmune disease that involves production of autoantibodies leading to inflammation-mediated tissue damages of many organs (2). While affecting both men and women, it appears to have a strong female bias. Characteristics of the disease include kidney inflammation, or lupus nephritis, and inflammation of the brain, among others. Leaky gut is also common in SLE patients (1). Recent research has demonstrated the importance of the gut microbiota in the disease pathogenesis, but to fully elucidate the interactions and mechanisms, more investigation is needed.

### Gut Microbiota

The composition of the gut microbiota has been shown to influence the diseased state. For example, the role of regulatory B (Breg) cells in SLE appears to be dependent on the disease stage, as prior to disease onset, B cells seem protective against induction, but later in the disease, B cells appear exacerbating (3). The latter finding is reasonable due to the cells’ ability to present autoantigens to T cells and produce autoantibodies (4, 5). The pre-disease protection by Breg cells is supported by vancomycin treatment resulting in a reduction in Breg cells and subsequent exacerbation of disease (3). Additionally, microbes appear to be involved in this protection, as oral supplementation with bacterial DNA can induce Breg cells and reduce autoimmunity (3).

Gavage studies again emphasize that the specific gut microbiota composition of lupus-prone mice contributes to disease. While female and male juvenile mouse gut microbiota is similar, distinctive differences arise in adulthood (6). Fecal transfer experiments suggest that the female microbiota promotes while the male microbiota slows the disease (6). There is an ongoing debate as to whether this altered gut microbiota composition is a cause or effect of SLE and whether it contributes to disease onset or worsens the active disease (3). While the aforementioned study relating bacterial DNA to disease suppression highlights the role of the microbiota in preventing disease initiation, C57BL/6 (B6) mice receiving fecal transfer from young lupus-prone B6.*Sle123* mice do not generate lupus-like symptoms (7). This may support that the gut microbiota acts as a disease exacerbator rather than initiator. In this case, a potential disease pathway has been suggested of genetic susceptibility leading to both autoimmunity and an altered microbiota, which in turn acts to worsen the disease. Similarly, the genetic background of the BXD2 lupus mouse model appeared to be responsible for disease initiation, while commensal bacterial affected its progression (8). Of note, the complex genetics of these mice is specifically designed to better represent human SLE. For ZAP70 mutated mice, the gut microbiota was not essential for disease initiation, but rather gut dysbiosis was essential for full disease onset (9).

Additionally, efforts continue to identify the most impactful species. In some studies, *Lactobacillus* has been shown to beneficially modulate disease, as gavage treatment of *Lactobacillus* spp. to the lupus-prone MRL/*lpr* mouse model improved symptoms and the intestinal barrier integrity (10). Interestingly, the benefit of *Lactobacillus* was only observed in female mice and when treatment was administered prior to disease onset. Hence, the gut microbiota may exert disease control in a sex- and time-dependent manner.

Further highlighting the gut microbiota, MRL/*lpr* mice treated with mixed antibiotics post-disease onset exhibited improved lupus-like symptoms along with an increase in *Lactobacillus* spp. but decrease in *Lachnospiraceae* (11). Some have thus considered *Lactobacillus* spp. to be “good” while *Lachnospiraceae* “bad”. Likewise, vancomycin treatment, which eliminates Gram-positive bacteria, but not *Lactobacillus*, replicated these results (11). These findings further the idea that *Lactobacillus* spp. may be an important part of the protective role that the gut microbiota could offer against SLE. However, the possibility that the decrease in harmful bacteria may instead be responsible for these results cannot be excluded.

Furthermore, it seems that gut dysbiosis can act as both a cause and an effect of autoimmunity. Genetic mutations in T cell receptor (TCR) signaling were shown to initiate autoimmunity, by promoting positive selection of self-reactive T cells and systemic T follicular helper (Tfh) cells as well as IgG production (9, 12, 13). Simultaneously, these mutations promoted gut dysbiosis through reduced positive selection for microbial-reactive T cells, leading to reduced gut Tfh cells and intestinal IgA (12). This altered gut microbiota then promotes Th17 cell development which furthers SLE development (12).

### Bacterial Translocation

Translocation of whole bacteria, including *Enterococcus gallinarum* and *Lactobacillus reuteri*, has been reported in mouse models and patients of SLE (14, 15). Bacterial components, such as lipopolysaccharide (LPS), can also affect lupus progression. LPS interacts with its receptor, toll-like receptor 4 (TLR4) whose activation has been shown to exacerbate lupus (1, 16–18). Dysfunction of the inhibitory receptor, Fc gamma receptor IIb (FcγRIIb) is observed in SLE and has been noted as a disease-causing agent (19, 20). Indeed, in mice with FcγRIIb knockout, translocated LPS and (1→3)-β-D-glucan resulted in much more severe inflammatory response (19). Additionally, gut leakage appears TLR7 dependent, since co-housing with lupus mice only resulted in leaky gut when the TLR7 gene was expressed (15).

### Bacterial Metabolites

The role of bacterial metabolites, such as short chain fatty acids (SCFAs) produced by colonic fermentation of resistant starch, in autoimmunity is another area of growing study. SCFAs are thought to have immunomodulatory effects after binding to their G-protein-coupled receptors, such as GPR43 (21–25). These metabolites can also affect gene transcription by inhibiting histone-deacetylase (HDAC) (22, 23). Some studies have shown SCFAs as inconsequential in protecting against or ameliorating lupus (3), while beneficial effects have been observed in the TLR7-dependent lupus model (15). Here, dietary supplementation of resistant starch produced SCFAs, ameliorated lupus and reduced *Lactobacillus reuteri* (15). More investigation is needed to determine whether SCFAs could be a dietary method to control lupus. Interestingly, while this study concluded that *Lactobacillus reuteri* alone contributed to lupus development for TLR7-dependent mice, others have found removal of *Lactobacillus* spp. harmful and supplementation beneficial for MRL/lpr and NZB/W F1 mice (3, 26). Thus, it is possible that effects of *Lactobacillus*, including amongst species and strains, vary between mouse models and may differ amongst subsets of SLE patients.

Gut microbes also contribute to tryptophan metabolism, which can produce immunomodulating molecules such as aryl hydrocarbon receptor ligands (27–29). Altered tryptophan metabolism has been observed for B6.*Sle123* mice, and low tryptophan diets reduced disease severity by preventing anti-dsDNA, improving kidney pathology, shifting the balance from Tfh towards T follicular regulatory (Tfr) cells, and enhancing Treg cells (7). However, *Lactobacillus* spp. is thought to be involved in leaky gut restoration, but since B6.*Sle123* mice do not exhibit leaky gut, probiotic treatment may not be beneficial. Indeed, high tryptophan diets worsened lupus symptoms and increased *Prevotellaceae, Paraprevotella and Lactobacillus* (7). Thus, it seems that dietary tryptophan and altered tryptophan metabolism is associated with gut microbiota dysbiosis, which can modulate lupus. Additionally, the effects of certain microbes again appear to rely heavily on the type of mouse model.

These studies highlight significant variations in microbiota-mediated disease mechanisms from one mouse model to another. The inconsistency among different mouse models may suggest diverse effects of gut microbiota on human SLE, where disease manifestations vary from patient to patient. A personalized approach toward treating SLE should be taken, with emphasis on acquiring a greater understanding of the pathogenesis for each disease manifestation.

## Type 1 Diabetes

### Background

Type 1 diabetes (T1D) is an autoimmune disease characterized by the destruction of insulin-producing β-cells in the pancreatic islets of Langerhans by self-reactive T cells (30, 31). While genetic components surely are involved, the growing incidence of T1D suggests a significant contribution from the environment (21). Appropriately, numerous intestinal changes have been linked to T1D, including changes to the gut microbiota, intestinal permeability, and intestinal inflammation (32, 33). Furthermore, the pancreas and intestines are likely lymphatically connected since the duodenal lymph node is synonymous with the pancreatic lymph node (PLN) (34). Since these lymph nodes drain both the pancreas and the duodenum, intestinal homeostatic changes can directly impact the pancreas and possibly contribute to the activation of islet-reactive T cells (35, 36). Of note, non-obese diabetic (NOD) mice are often used to model human T1D since they develop disease similarly with autoantibodies and elevated circulating T cells before T1D onset (37, 38).

### Gut Microbiota

Of the two major intestinal barriers, the intestinal epithelial barrier and the mucus layer, the permeability of the intestinal epithelial barrier, often involving reduced or dysfunctional tight junction proteins, has primarily been associated with T1D (39). However, mucus layer alterations, including thinning, breakage, reduced goblet cells and mucus production, and shift toward proinflammatory mucin expression, have been observed in NOD mice (39). As such, these mice were observed to have increased intestinal permeability; but interestingly, no change in structural proteins. Intestinal permeability could act as a T1D trigger by facilitating bacterial component leakage, activating mucosal T cells which subsequently migrate to the PLNs and islets. However, to induce islet-reactive T cells, the presence of gut commensal bacteria appears to be required (39). The specific composition of commensal bacteria appears critical, as maternal NOR microbiota transmission, used as a control for NOD mice, to newborn NOD mice altered their commensal bacteria and prevented T1D (34). Further, cross-fostering successfully restored the mucus barrier and normalized goblet cell levels (34). The reduction in secretory IgA (SIgA) in NOD mice could be a reason for their altered bacterial composition, since SIgA contributes to commensal bacteria shaping (40). Thus, altered commensal bacteria and intestinal barrier dysfunction, could together act as T1D triggers. This is supported by these features and additional proinflammatory intestinal alterations manifesting at an age well before disease onset (34).

Treg cells in the intestine that produce IL-10 can migrate and regulate effector T cells, offering protection against T1D (41). As observed after probiotic supplementation, the gut microbiota also appears to be capable of inducing IL-10 producing cells in the gut-associated lymphoid tissue and prevent T1D (41). Furthermore, a probiotic treatment of *Lactobacillus acidophilus, Lactobacillus casei, Lactobacillus reuteri, Bifidobacterium bifidium*, and *Streptococcus thermophiles* (called Immune Regulation and Tolerance 5, IRT5) has shown to at least offer partial protection against T1D (42). This appeared to operate through enhancing Treg homing in the gut, by expressing the gut-homing receptor CCR9, reducing IFN-γ producing T helper (Th1) cells, and reducing intestinal permeability (42).

Polymorphisms of the major histocompatibility (MHC) locus are a genetic feature of both human and murine T1D (31, 43). While NOD mice inherently lack the *Eα* complex of the MHC-II protein, genetic modifications leading to its expression prevent T1D (44–47). The microbiota may be involved in this protection as microbiota differences of *Eα*-expressing and NOD mice appears at an early age, approximately when the intestines gain lymphatic connection to the pancreas, and self-antigens are seen in the PLN (31). Maternal expression of the *Eα* complex seems important, as their progeny experienced reduced T1D incidence and insulitis (31). Notably, this expression only seems protective when the maternal microbiota is intact, as some progeny developed insulitis after maternal antibiotic treatment (31). Furthermore, germ-free *Eα*-expressing NOD mice developed insulitis to similar extents and severity as NOD mice and germ-free NOD mice (31). On a translational level, this may suggest that antibiotics to mothers and infants could be detrimental by preventing the gut microbiota from offering protection against autoimmunity.

### Bacterial Metabolites

In further support of the involvement of the gut bacterial composition in T1D is that mice deficient in the signaling molecule MyD88 are protected from T1D only when housed in non-germ-free conditions (21, 48). Knockout of MyD88 results in the over-representation of *Bacteroidetes*, which could exert protection from T1D due to their production of SCFAs (22). Direct dietary supplementation suggests benefits of high levels the SCFAs acetone and butyrate (21). While independently, these diets offered only partial protection from T1D, in combination they offered complete protection and reduced leaky gut in NOD mice (21). Therefore, either supporting the bacteria that supply these SCFAs or supplementing directly with SCFAs could potentially mitigating T1D. However, the possibility that other bacterial metabolites may worsen or contribute to T1D onset still should be considered.

## Multiple Sclerosis

### Background

Multiple sclerosis (MS) is an inflammatory autoimmune disease of the central nervous system (CNS). T cells become reactive against myelin self-antigens, creating a pro-inflammatory environment, and facilitating demyelination and neurodegeneration (49, 50). This appears to involve the differentiation of T cells into Th1 and Th17 cells, and their subsequent excessive production and release of pro-inflammatory cytokines IFNγ and IL-17, as they roam about the CNS (51, 52). While genetics contribute to the disease, the importance of the environment and interactions between genetic factors and the environment should not be undervalued (53–55).

### Gut Microbiota

The gut microbiota has been noted as involved in MS pathogenesis (55–57). Indeed, both MS patients and mouse models induced with experimental autoimmune encephalomyelitis (EAE), commonly used to study MS, experience gut dysbiosis (58–62). For instance, in EAE induced B6 mice, the relative abundance of certain bacteria appears to be upregulated (such as *Proteobacteria* and *Deferribacteres*) while others downregulated (such as *Bacteroidetes*) (62). However, probiotic treatment with *Lactobacillus reuteri* reshaped the microbiota back towards that of the control, and reduced CNS immune cell abundance, infiltration, and abundance of inflammatory cytokines (62). Overall, it seems that interactions between gut microbes may be more impactful in dictating EAE severity rather than individual microbial species (55).

Interestingly, both probiotic *Clostridium butyricum* treatment and antibiotic norfloxacin fluoroquinolone treatment have been successful in ameliorating EAE in B6 mice despite their contradictory roles (63). While both treatment groups resulted in the expected change in bacterial load and distinct microbiota clustering, they also both resulted in a similar ratio of high *Bacteroidetes* to low *Firmicutes* (63). These microbial changes were also observed to alter T cell differentiation away from Th17 cells and towards Treg cells, possibly by reducing the activity of p38 mitogen activated protein kinase (MAPK) and c-Jun N-terminal Kinase (JNK) pathways (63).

Furthermore, while IL-17 is often associated with having a pro-inflammatory role, it appears to be more directly involved in shaping the gut microbiota which in turn affects the disease state. IL–17A and IL-17F double knockout mice exhibited a gut microbiota significantly different from wild type B6 mice and were protected from developing EAE (64). Reintroducing IL-17A expression specifically in the gut epithelium using Cre recombinase expression by the *Villin1* promoter, altered the microbiota and promoted EAE susceptibility (64).

Overall, the recent literature advances our understanding of how the gut microbiota regulates CNS inflammation and vice versa, revealing mechanisms to support the efficacy of new treatment strategies, such as fecal microbiota transplantation (65), which is currently being tested for the treatment of MS in clinical trials.

Despite the inclination to associate certain bacteria with predetermined disease effects, the impact of the genetic background makes this association not so straightforward. For mice genetically susceptible to EAE, *Lactobacillus reuteri* independently exacerbated the disease, but interestingly this strain was in high abundance for the disease-resistant wild-derived inbred strain, PWD/PhJ (PWD) (55). Correspondingly, microbiota transplant from PWD mice worsened disease in EAE prone mice (55). Also of note, *Lactobacillus reuteri* ameliorated disease in EAE induced B6 mice (62). These results suggest that microbial species can have differential effects depending on the host’s genetic background, which will be a crucial consideration when investigating probiotic therapies for autoimmune diseases.

Additionally, in MS, certain human leukocyte antigen (HLA) class II haplotypes have been observed to be especially prominent, which is important since HLA class II molecules are involved in CD4 T cell selection processes (66, 67). Hence, it is valuable to mention that disease-susceptible transgenic mice expressing human HLA-DR3 and DQ8 genes have disease suppression or reduced severity after treatment with *Prevotella histicola* (68). Not only was this treatment shown to restore the gut barrier, but also the blood brain barrier, coupled with reduced migration of inflammatory T cells to the CNS. Additionally, this treatment induced Treg cells and enhanced their suppressive function, while downregulating Th1 and Th17 cells and their respective cytokines (68). This again supports the view that bacterial treatment could be a therapeutic option for autoimmune disease, pending appropriate genetic background consideration.

### Intestinal Permeability

For EAE induced B6 mice, increased intestinal permeability before clinical disease onset has been observed, along with downregulated expression of antimicrobial peptides and tight junction proteins, which are crucial for the mucus layer and intestinal epithelial layer, respectively (69). These deleterious features were reduced after probiotic treatment using *E. coli* Nissle 1917, supporting the notion that microbial composition can directly impact gut leakiness and subsequently promote or inhibit disease.

## Discussion

**Figure 1:**
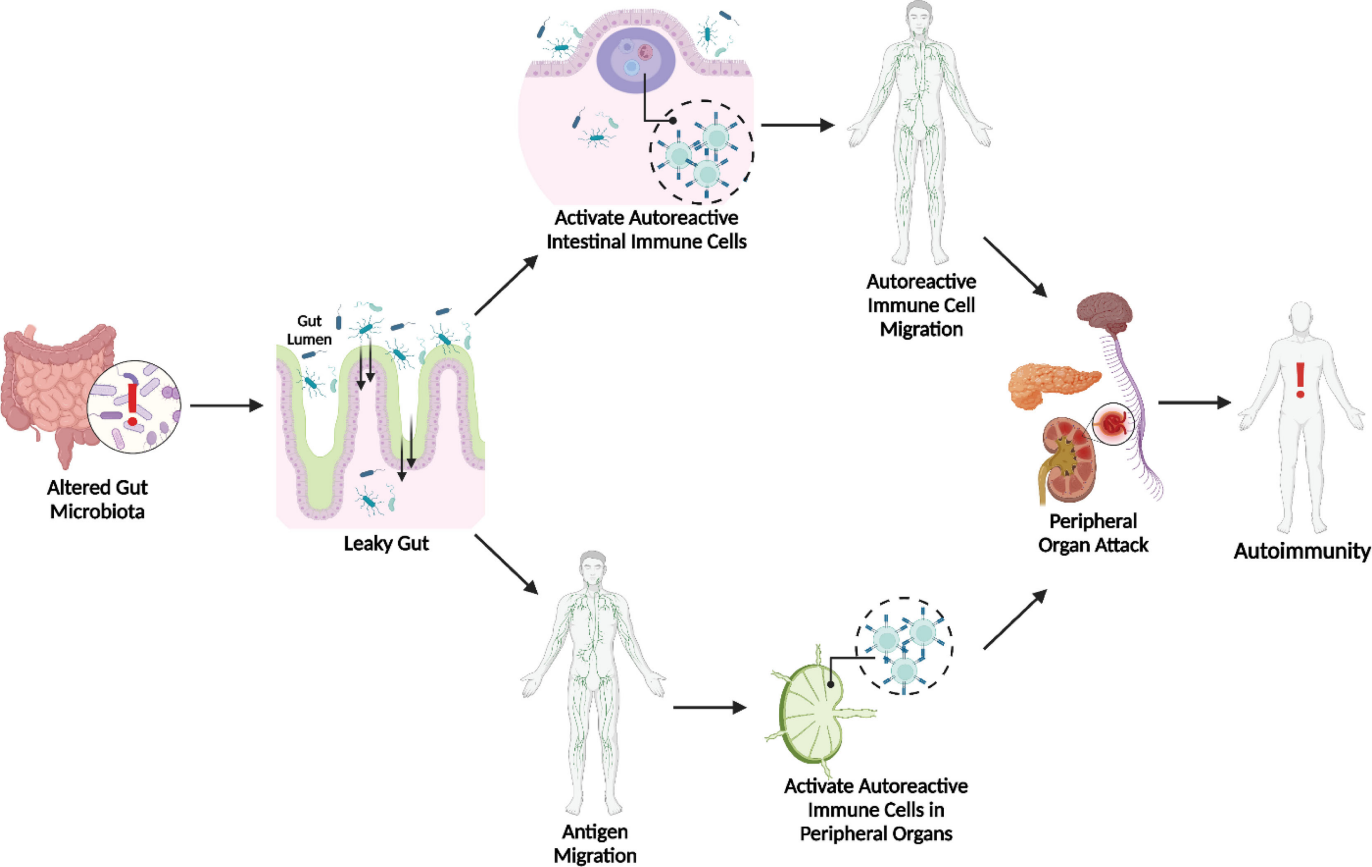
Microbial Dysbiosis & Leaky Gut Initiated Pathways to Autoimmunity. Both activated autoreactive intestinal immune cell translocation (35) and bacterial antigen translocation (14–19, 70) to peripheral sites can lead to autoimmunity.

Overall, the gut microbiota appears closely involved in autoimmune pathogenesis, likely due to its ability to alter the intestinal barrier. In the modern world, this realization is critical since microbial dysbiosis is very obtainable *via* the Western diet, antibiotic usage, and excessive sanitation. As demonstrated in **Figure 1**, this gut dysbiosis could trigger autoimmune disease through two potential pathways. After promoting a leaky gut, bacterial antigens could stimulate intestinal immune cells, generating autoreactive cells that subsequently migrate systemically to their target peripheral organs and initiate attack. However, bacterial antigens themselves could migrate systemically and generate autoreactive immune cells within the lymphatic connections of the peripheral organs. Although the environmental ties to autoimmunity are concerning, these findings offer many routes for studying prospective therapies. It is important to acknowledge that it may be impetuous to classify bacteria as solely “good” or “bad” because of the important interactions between and dependence on the host’s genetics and pre-established microbiota. Hence, both probiotics and antibiotics could be potential treatments. Much more investigation is needed to identify the specific mechanisms and interactions between the microbiota and genetic background to properly design host-specific therapies. Furthermore, dietary interventions, such as a high fiber diet facilitating SCFAs may be promising due to research suggesting their ability to affect immune cell differentiation. Overall, the findings presented here on the contributions of the gut microbiota to SLE, T1D, and MS represent the great strides taken towards advancing the understanding of the role of the environment in autoimmunity. While much still needs to be discovered, current research is paving the way for novel clinical interventions to better manage or prevent these life-altering conditions.

## Author Contributions

AC did the literature search and wrote the manuscript. XL edited the manuscript. All authors contributed to the article and approved the submitted version.

## Conflict of Interest

The authors declare that the research was conducted in the absence of any commercial or financial relationships that could be construed as a potential conflict of interest.

## Publisher’s Note

All claims expressed in this article are solely those of the authors and do not necessarily represent those of their affiliated organizations, or those of the publisher, the editors and the reviewers. Any product that may be evaluated in this article, or claim that may be made by its manufacturer, is not guaranteed or endorsed by the publisher.

## References

[1] Mu Q Kirby J Reilly CM Luo XM. Leaky Gut as a Danger Signal for Autoimmune Diseases. Front Immunol (2017) 8:598. doi: 10.3389/fimmu.2017.00598 28588585 PMC5440529

[2] Tsokos GC. Mechanisms of Disease: Systemic Lupus Erythematosus. New Engl J Med (2011) 365:2110. doi: 10.1056/nejmra1100359 22129255

[3] Mu Q Edwards MR Swartwout BK Cabana Puig X Mao J Zhu J. Gut Microbiota and Bacterial DNA Suppress Autoimmunity by Stimulating Regulatory B Cells in a Murine Model of Lupus. Front Immunol (2020) 11:593353. doi: 10.3389/fimmu.2020.593353 33240280 PMC7683516

[4] Jacob N Stohl W. Autoantibody-Dependent and Autoantibody-Independent Roles for B Cells in Systemic Lupus Erythematosus: Past, Present, and Future. Autoimmunity (2010) 43:84. doi: 10.3109/08916930903374600 20014977 PMC2809122

[5] Dörner T Giesecke C Lipsky PE. Mechanisms of B Cell Autoimmunity in SLE. Arthritis Res Ther (2011) 13:243. doi: 10.1186/ar3433 22078750 PMC3308063

[6] Johnson BM Gaudreau MC Gudi R Brown R Gilkeson G Vasu C. Gut Microbiota Differently Contributes to Intestinal Immune Phenotype and Systemic Autoimmune Progression in Female and Male Lupus-Prone Mice. J Autoimmun (2020) 108:102420. doi: 10.1016/j.jaut.2020.102420 32019684 PMC7204266

[7] Choi SC Brown J Gong M Ge Y Zadeh M Li W. Gut Microbiota Dysbiosis and Altered Tryptophan Catabolism Contribute to Autoimmunity in Lupus-Susceptible Mice. Sci Trans Med (2020) 12:eaax2220. doi: 10.1126/SCITRANSLMED.AAX2220 PMC7739186 32641487

[8] Hong H Alduraibi F Ponder D Duck WL Morrow CD Foote JB. Host Genetics But Not Commensal Microbiota Determines the Initial Development of Systemic Autoimmune Disease in BXD2 Mice. Arthritis Rheumatol (2022) 74:634. doi: 10.1002/art.42008 34725967 PMC9071869

[9] Matsuo T Hashimoto M Sakaguchi S Sakaguchi N Ito Y Hikida M. Strain-Specific Manifestation of Lupus-Like Systemic Autoimmunity Caused by Zap70 Mutation. J Immunol (2019) 202:631. doi: 10.4049/jimmunol.1801159 31019063

[10] Mu Q Zhang H Liao X Lin K Liu H Edwards MR. Control of Lupus Nephritis by Changes of Gut Microbiota. Microbiome (2017) 5:73. doi: 10.1186/s40168-017-0300-8 28697806 PMC5505136

[11] Mu Q Tavella VJ Kirby JL Cecere TE Chung M Lee J. Antibiotics Ameliorate Lupus-Like Symptoms in Mice. Sci Rep (2017) 7:13575. doi: 10.1038/s41598-017-14223-0 29057975 PMC5651817

[12] Shirakashi M Maruya M Hirota K Tsuruyama T Matsuo T Watanabe R. Effect of Impaired T Cell Receptor Signaling on the Gut Microbiota in a Mouse Model of Systemic Autoimmunity. Arthritis Rheumatol (2022) 74:641. doi: 10.1002/art.42016 34725966

[13] Sakaguchi N Takahashi T Hata H Nomura T Tagami T Yamazaki S. Altered Thymic T-Cell Selection Due to a Mutation of the ZAP-70 Gene Causes Autoimmune Arthritis in Mice. Nature (2003) 426:454. doi: 10.1038/nature02119 14647385

[14] Manfredo Vieira S Hiltensperger M Kumar V Zegarra-Ruiz D Dehner C Khan N. Translocation of a Gut Pathobiont Drives Autoimmunity in Mice and Humans. Science (2018) 359:1156. doi: 10.1126/science.aar7201 29590047 PMC5959731

[15] Zegarra-Ruiz DF El Beidaq A Iniguez AJ Lubrano Di Ricco M Manfredo Vieira S Ruff WE. A Diet-Sensitive Commensal Lactobacillus Strain Mediates TLR7-Dependent Systemic Autoimmunity. Cell Host Microbe (2019) 25:113. doi: 10.1016/j.chom.2018.11.009 30581114 PMC6377154

[16] Lee TP Huang JC Liu CJ Chen HJ Chen YH Tsai YT. Featured Article: Interactions of Surface-Expressed TLR-4 and Endosomal TLR-9 Accelerate Lupus Progression in anti-dsDNA Antibody Transgenic Mice. Exp Biol Med (2014) 239:715. doi: 10.1177/1535370214525299 24719374

[17] Lee TP Tang SJ Wu MF Song YC Yu CL Sun KH. Transgenic Overexpression of Anti-Double-Stranded DNA Autoantibody and Activation of Toll-Like Receptor 4 in Mice Induce Severe Systemic Lupus Erythematosus Syndromes. J Autoimmun (2010) 35:358. doi: 10.1016/j.jaut.2010.07.007 20833510

[18] Liu B Yang Y Dai J Medzhitov R Freudenberg MA Zhang PL. TLR4 Up-Regulation at Protein or Gene Level Is Pathogenic for Lupus-Like Autoimmune Disease. J Immunol (2006) 177:6880. doi: 10.4049/jimmunol.177.10.6880 17082602

[19] Thim-uam A Surawut S Issara-Amphorn J Jaroonwitchawan T Hiengrach P Chatthanathon P. Leaky-Gut Enhanced Lupus Progression in the Fc Gamma Receptor-IIb Deficient and Pristane-Induced Mouse Models of Lupus. Sci Rep (2020) 10:777. doi: 10.1038/s41598-019-57275-0 31964918 PMC6972921

[20] Bolland S Ravetch J v. Spontaneous Autoimmune Disease in Fcγriib-Deficient Mice Results From Strain-Specific Epistasis. Immunity (2000) 13:277. doi: 10.1016/S1074-7613(00)00027-3 10981970

[21] Mariño E Richards JL McLeod KH Stanley D Yap YA Knight J. Gut Microbial Metabolites Limit the Frequency of Autoimmune T Cells and Protect Against Type 1 Diabetes. Nat Immunol (2017) 18:552. doi: 10.1038/ni.3713 28346408

[22] Thorburn AN Macia L Mackay CR. Diet, Metabolites, and “Western-Lifestyle” Inflammatory Diseases. Immunity (2014) 40:833. doi: 10.1016/j.immuni.2014.05.014 24950203

[23] Maslowski KM Vieira AT Ng A Kranich J Sierro F Yu di. Regulation of Inflammatory Responses by Gut Microbiota and Chemoattractant Receptor GPR43. Nature (2009) 461:1282. doi: 10.1038/nature08530 19865172 PMC3256734

[24] Smith PM Howitt MR Panikov N Michaud M Gallini CA Bohlooly-Y M. The Microbial Metabolites, Short-Chain Fatty Acids, Regulate Colonic T Reg Cell Homeostasis. Science (1979) (2013) 341:569. doi: 10.1126/science.1241165 PMC3807819 23828891

[25] Furusawa Y Obata Y Fukuda S Endo TA Nakato G Takahashi D. Commensal Microbe-Derived Butyrate Induces the Differentiation of Colonic Regulatory T Cells. Nature (2013) 504:466. doi: 10.1038/nature12721 24226770

[26] Zhang H Liao X Sparks JB Luo XM. Dynamics of Gut Microbiota in Autoimmune Lupus. Appl Environ Microbiol (2014) 80:7551. doi: 10.1128/AEM.02686-14 25261516 PMC4249226

[27] Cervantes-Barragan L Chai JN Tianero MD di Luccia B Ahern PP Merriman J. Lactobacillus Reuteri Induces Gut Intraepithelial CD4+CD8αα+ T Cells. Science (1979) (2017) 357:806. doi: 10.1126/science.aah5825 PMC5687812 28775213

[28] Dodd D Spitzer MH van Treuren W Merrill BD Hryckowian AJ Higginbottom SK. A Gut Bacterial Pathway Metabolizes Aromatic Amino Acids Into Nine Circulating Metabolites. Nature (2017) 551:648. doi: 10.1038/nature24661 29168502 PMC5850949

[29] Rothhammer V Mascanfroni ID Bunse L Takenaka MC Kenison JE Mayo L. Type I Interferons and Microbial Metabolites of Tryptophan Modulate Astrocyte Activity and Central Nervous System Inflammation *via* the Aryl Hydrocarbon Receptor. Nat Med (2016) 22:586. doi: 10.1038/nm.4106 27158906 PMC4899206

[30] Todd JA. Etiology of Type 1 Diabetes. Immunity (2010) 32:457. doi: 10.1016/j.immuni.2010.04.001 20412756

[31] Silverman M Kua L Tanca A Pala M Palomba A Tanes C. Protective Major Histocompatibility Complex Allele Prevents Type 1 Diabetes by Shaping the Intestinal Microbiota Early in Ontogeny. Proc Natl Acad Sci U S A (2017) 114:9671. doi: 10.1073/pnas.1712280114 28831005 PMC5594701

[32] Badami E Sorini C Coccia M Usuelli V Molteni L Bolla AM. Defective Differentiation of Regulatory FoxP3+ T Cells by Small-Intestinal Dendritic Cells in Patients With Type 1 Diabetes. Diabetes (2011) 60:2120. doi: 10.2337/db10-1201 21646390 PMC3142071

[33] Vatanen T Franzosa EA Schwager R Tripathi S Arthur TD Vehik K. The Human Gut Microbiome in Early-Onset Type 1 Diabetes From the TEDDY Study. Nature (2018) 562:589. doi: 10.1038/s41586-018-0620-2 30356183 PMC6296767

[34] Miranda MCG Oliveira RP Torres L Aguiar SLF Pinheiro-Rosa N Lemos L. Frontline Science: Abnormalities in the Gut Mucosa of non-Obese Diabetic Mice Precede the Onset of Type 1 Diabetes. J Leukocyte Biol (2019) 106:513. doi: 10.1002/JLB.3HI0119-024RR 31313381

[35] Turley SJ Lee JW Dutton-Swain N Mathis D Benoist C. Endocrine Self and Gut non-Self Intersect in the Pancreatic Lymph Nodes. Proc Natl Acad Sci U S A (2005) 102:17729. doi: 10.1073/pnas.0509006102 16317068 PMC1308925

[36] van den Broeck W Derore A Simoens P. Anatomy and Nomenclature of Murine Lymph Nodes: Descriptive Study and Nomenclatory Standardization in BALB/cAnNCrl Mice. J Immunol Methods (2006) 312:12. doi: 10.1016/j.jim.2006.01.022 16624319

[37] Gregori S Giarratana N Smiroldo S Adorini L. Dynamics of Pathogenic and Suppressor T Cells in Autoimmune Diabetes Development. J Immunol (2003) 171:4040. doi: 10.4049/jimmunol.171.8.4040 14530324

[38] Wicker LS Clark J Fraser HI Garner VES Gonzalez-Munoz A Healy B. Type 1 Diabetes Genes and Pathways Shared by Humans and NOD Mice. J Autoimmun (2005) 25(Suppl.29). doi: 10.1016/j.jaut.2005.09.009 16257508

[39] Sorini C Cosorich I Lo Conte M de Giorgi L Facciotti F Lucianò R. Loss of Gut Barrier Integrity Triggers Activation of Islet-Reactive T Cells and Autoimmune Diabetes. Proc Natl Acad Sci U S A (2019) 116:15140. doi: 10.1073/pnas.1814558116 31182588 PMC6660755

[40] Rollenske T Burkhalter S Muerner L von Gunten S Lukasiewicz J Wardemann H. Parallelism of Intestinal Secretory IgA Shapes Functional Microbial Fitness. Nature (2021) 598:657. doi: 10.1038/s41586-021-03973-7 34646015

[41] Yu H Gagliani N Ishigame H Huber S Zhu S Esplugues E. Intestinal Type 1 Regulatory T Cells Migrate to Periphery to Suppress Diabetogenic T Cells and Prevent Diabetes Development. Proc Natl Acad Sci U S A (2017) 114:10443. doi: 10.1073/pnas.1705599114 28894001 PMC5625908

[42] Kim TK Lee JC Im SH Lee MS. Amelioration of Autoimmune Diabetes of NOD Mice by Immunomodulating Probiotics. Front Immunol (2020) 11:1832. doi: 10.3389/fimmu.2020.01832 33013834 PMC7496355

[43] Noble JA Valdes AM. Genetics of the HLA Region in the Prediction of Type 1 Diabetes. Curr Diabetes Rep (2011) 11:533. doi: 10.1007/s11892-011-0223-x PMC3233362 21912932

[44] Böhme J Schuhbaur B Kanagawa O Benoist C Mathis D. MHC-Linked Protection From Diabetes Dissociated From Clonal Deletion of T Cells. Science (1979) (1990) 249:293. doi: 10.1126/science.2115690 2115690

[45] Lund T O’Reilly L Hutchings P Kanagawa O Simpson E Gravely R. Prevention of Insulin-Dependent Diabetes Mellitus in non-Obese Diabetic Mice by Transgenes Encoding Modified I-A β-Chain or Normal I-E α-Chain. Nature (1990) 345:727. doi: 10.1038/345727a0 2163026

[46] Mathis DJ Benoist C Williams VE Kanter M McDevitt HO. Several Mechanisms can Account for Defective E Alpha Gene Expression in Different Mouse Haplotypes. Proc Natl Acad Sci U S A (1983) 80:273. doi: 10.1073/pnas.80.1.273 6296871 PMC393355

[47] Nishimoto H Kikutani H Yamamura KI Kishimoto T. Prevention of Autoimmune Insulitis by Expression of I-E Molecules in NOD Mice. Nature (1988) 328:432. doi: 10.1038/328432a0 3302721

[48] Wen L Ley RE Volchkov PY Stranges PB Avanesyan L Stonebraker AC. Innate Immunity and Intestinal Microbiota in the Development of Type 1 Diabetes. Nature (2008) 455:1109. doi: 10.1038/nature07336 18806780 PMC2574766

[49] Sospedra M Martin R. Immunology of Multiple Sclerosis. Semin Neurol (2016) 36:115. doi: 10.1055/s-0036-1579739 27116718

[50] Cao Y Goods BA Raddassi K Nepom GT Kwok WW Love JC. Functional Inflammatory Profiles Distinguish Myelin-Reactive T Cells From Patients With Multiple Sclerosis. Sci Trans Med (2015) 7:287ra74. doi: 10.1126/scitranslmed.aaa8038 PMC4497538 25972006

[51] Dendrou CA Fugger L Friese MA. Immunopathology of Multiple Sclerosis. Nat Rev Immunol (2015) 15:545. doi: 10.1038/nri3871 26250739

[52] McFarland HF Martin R. Multiple Sclerosis: A Complicated Picture of Autoimmunity. Nat Immunol (2007) 8:913. doi: 10.1038/ni1507 17712344

[53] Beecham AH Patsopoulos NA Xifara DK Davis MF Kemppinen A Cotsapas C. Analysis of Immune-Related Loci Identifies 48 New Susceptibility Variants for Multiple Sclerosis. Nat Genet (2013) 45:1353. doi: 10.1038/ng.2770 24076602 PMC3832895

[54] Ebers GC. Environmental Factors and Multiple Sclerosis. Lancet Neurol (2008) 7:268. doi: 10.1016/S1474-4422(08)70042-5 18275928

[55] Montgomery TL Künstner A Kennedy JJ Fang Q Asarian L Culp-Hill R. Interactions Between Host Genetics and Gut Microbiota Determine Susceptibility to CNS Autoimmunity. Proc Natl Acad Sci U S A (2020) 117:27516. doi: 10.1073/pnas.2002817117 33077601 PMC7959502

[56] Belkaid Y Hand TW. Role of the Microbiota in Immunity and Inflammation. Cell (2014) 157:121. doi: 10.1016/j.cell.2014.03.011 24679531 PMC4056765

[57] Grigg JB Sonnenberg GF. Host-Microbiota Interactions Shape Local and Systemic Inflammatory Diseases. J Immunol (2017) 198:564. doi: 10.4049/jimmunol.1601621 28069751 PMC5228396

[58] Berer K Mues M Koutrolos M AlRasbi Z Boziki M Johner C. Commensal Microbiota and Myelin Autoantigen Cooperate to Trigger Autoimmune Demyelination. Nature (2011) 479:28484. doi: 10.1038/nature10554 22031325

[59] Chen J Chia N Kalari KR Yao JZ Novotna M Soldan MMP. Multiple Sclerosis Patients Have a Distinct Gut Microbiota Compared to Healthy Controls. Sci Rep (2016) 6. doi: 10.1038/srep28484 PMC4921909 27346372

[60] Jangi S Gandhi R Cox LM Li N von Glehn F Yan R. Alterations of the Human Gut Microbiome in Multiple Sclerosis. Nat Commun (2016) 7:12015. doi: 10.1038/ncomms12015 27352007 PMC4931233

[61] Newland PK Heitkemper M Zhou Y. The Emerging Role of the Gut Microbiome in Adult Patients With Multiple Sclerosis. J Neurosci Nurs (2016) 48:358. doi: 10.1097/JNN.0000000000000252 27824805

[62] He B Hoang TK Tian X Taylor CM Blanchard E Luo M. Lactobacillus Reuteri Reduces the Severity of Experimental Autoimmune Encephalomyelitis in Mice by Modulating Gut Microbiota. Front Immunol (2019) 10:385. doi: 10.3389/fimmu.2019.00385 30899262 PMC6416370

[63] Chen H Ma X Liu Y Ma L Chen Z Lin X. Gut Microbiota Interventions With Clostridium Butyricum and Norfloxacin Modulate Immune Response in Experimental Autoimmune Encephalomyelitis Mice. Front Immunol (2019) 10:1662. doi: 10.3389/fimmu.2019.01662 31428083 PMC6689973

[64] Regen T Isaac S Amorim A Núñez NG Hauptmann J Shanmugavadivu A. IL-17 Controls Central Nervous System Autoimmunity Through the Intestinal Microbiome. Sci Immunol (2021) 6:eaaz6563. doi: 10.1126/SCIIMMUNOL.AAZ6563 33547052

[65] Engen PA Zaferiou A Rasmussen H Naqib A Green SJ Fogg LF. Single-Arm, non-Randomized, Time Series, Single-Subject Study of Fecal Microbiota Transplantation in Multiple Sclerosis. Front Neurol (2020) 11:978. doi: 10.3389/fneur.2020.00978 33013647 PMC7506051

[66] Zivadinov R Uxa L Bratina A Bosco A Srinivasaraghavan B Minagar A. HLA-DRB1*1501, -DQB1*0301, -DQB1*0302, -DQB1*0602, and -DQB1*0603 Alleles are Associated With More Severe Disease Outcome on Mri in Patients With Multiple Sclerosis. Int Rev Neurobiol (2007) 79:521. doi: 10.1016/S0074-7742(07)79023-2 17531857

[67] Dyment DA Herrera BM Cader MZ Willer CJ Lincoln MR Sadovnick AD. Complex Interactions Among MHC Haplotypes in Multiple Sclerosis: Susceptibility and Resistance. Hum Mol Genet (2005) 14:2019. doi: 10.1093/hmg/ddi206 15930013

[68] Mangalam A Shahi SK Luckey D Karau M Marietta E Luo N. Human Gut-Derived Commensal Bacteria Suppress CNS Inflammatory and Demyelinating Disease. Cell Rep (2017) 20:1269. doi: 10.1016/j.celrep.2017.07.031 28793252 PMC5763484

[69] Secher T Kassem S Benamar M Bernard I Boury M Barreau F. Oral Administration of the Probiotic Strain Escherichia Coli Nissle 1917 Reduces Susceptibility to Neuroinflammation and Repairs Experimental Autoimmune Encephalomyelitis-Induced Intestinal Barrier Dysfunction. Front Immunol (2017) 8:1096. doi: 10.3389/fimmu.2017.01096 28959254 PMC5603654

[70] Sun J Furio L Mecheri R van der Does AM Lundeberg E Saveanu L. Pancreatic β-Cells Limit Autoimmune Diabetes *via* an Immunoregulatory Antimicrobial Peptide Expressed Under the Influence of the Gut Microbiota. Immunity (2015) 43. doi: 10.1016/j.immuni.2015.07.013 26253786

